# Traumatic Globe Enucleation After Blunt Head Injury

**DOI:** 10.7759/cureus.24476

**Published:** 2022-04-25

**Authors:** Shahriyar Shafa, Amin Zand, Ali Sharifi, Mahdi Sharifzadeh

**Affiliations:** 1 Ophthalmology, Shafa Hospital, Kerman University of Medical Sciences, Kerman, IRN

**Keywords:** traumatic globe avulsion, auto enucleation, globe avulsion, globe luxation, traumatic enucleation

## Abstract

We report a 57-year-old female with left globe enucleation following head trauma after falling. The left globe was intact and protruded from the orbit. A CT scan revealed anterior globe protrusion with avulsions of the optic nerve and the extraocular muscles with posterior-lateral dislocation of the left lateral orbital wall. Due to the unstable general condition (with signs of intracranial hemorrhage), the patient was admitted to the ICU, and the removal of the completely avulsed globe was postponed. After stabilizing the general condition, the avulsed globe with adjacent structures including a part of the optic nerve was removed in the operation theater and the patient was planned for future orbital reconstructive surgeries including ocular prosthesis. The patient underwent close follow-up visits during the admission for detecting any signs of sympathetic ophthalmia progression in the fellow eye. Traumatic enucleation is a rare condition and can be caused not only by direct and high-energy traumas, but also by the indirect mode of trauma with no significant orbital wall disassembly. In these patients, predisposition to globe luxation must be considered and advised for protecting the fellow eye from any traumas.

## Introduction

The auto-enucleation of the globe is mainly prevalent in psychiatric or drug-abuse patients [1,2]. Traumatic enucleation is less common, and only a few reports of this condition exist [3-12]. In most reports, patients experienced orbital fractures with maxillofacial fractures during high-energy traumas [5,8-12]. Here, we report a patient with traumatic enucleation following a head injury after falling without significant disassembly in orbital walls, as a rare presentation of traumatic enucleation.

## Case presentation

**Figure 1:**
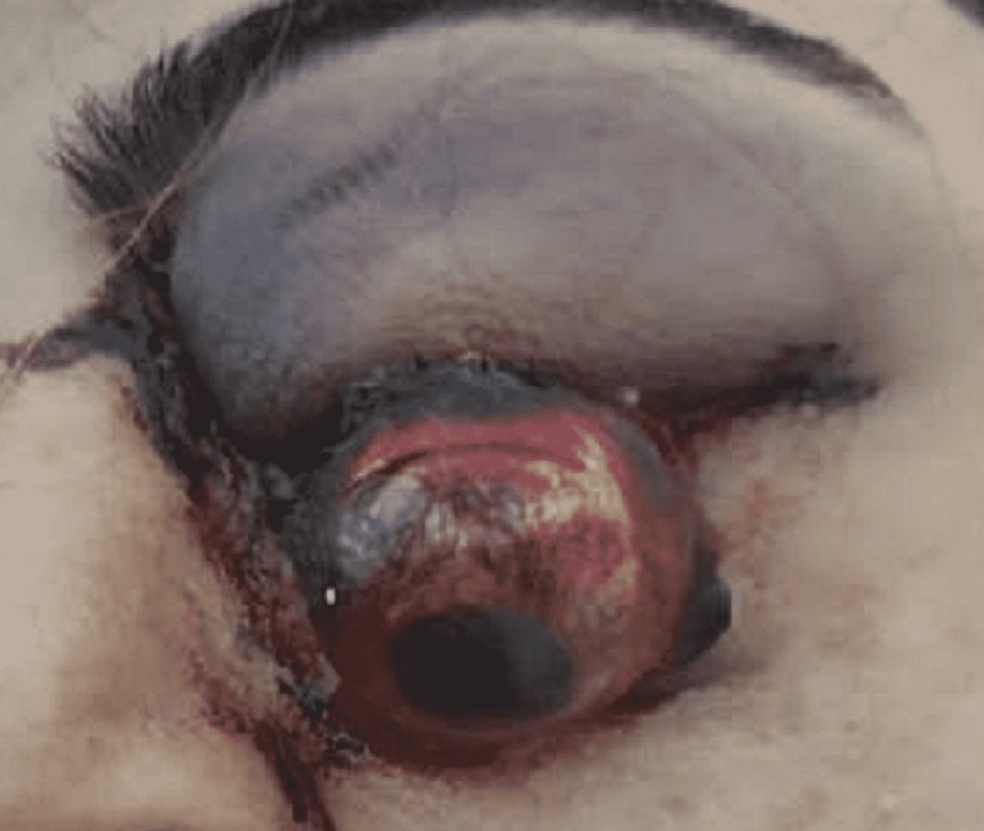
Total overhanging of the left globe from the orbit. The globe did not have any obvious lacerations

The patient was a 57-year-old female who had suffered blunt trauma to the head (especially on the left temporal side) during an accidental falling down onto the edge of the bed corner the previous day. She was semiconscious (Glasgow Coma Scale - GCS: 11/15) with stable vital signs. She had a left facial soft tissue injury with a hematoma at the left temporal side of her head. The left globe protruded almost completely outside the orbit. The protruded globe did not have any obvious lacerations (Figure 1).

**Figure 2:**
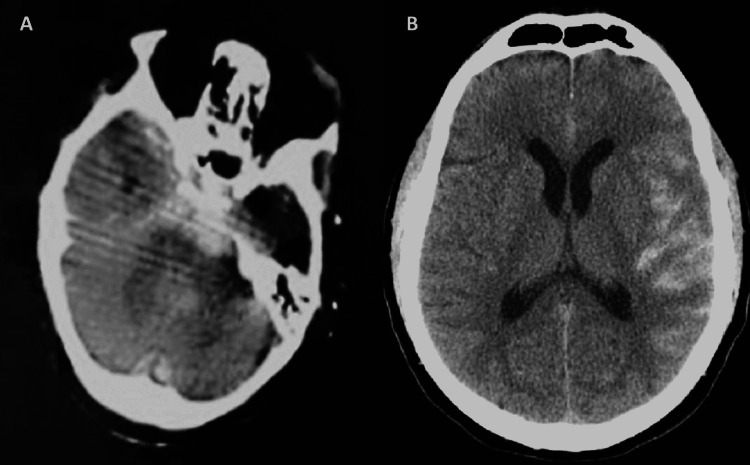
(A) Axial orbital CT scan showed left lateral orbital wall dislocation to the posterior-lateral side. (B) Axial brain CT scan revealed a left-sided subarachnoid hemorrhage CT, computed tomography.

Due to her general condition, assessment of visual acuity was not possible. Left eye examination revealed diffuse bloody chemosis, severe corneal edema, macroscopic hyphema, fixed and dilated pupil without any red reflex, and fundus was invisible. Further examination of the right eye was normal. Orbital CT scans showed traumatic enucleation of the left eye. The globe was intact and completely avulsed from other structures, including the optic nerve, and extraocular muscles with complete anterior globe protrusion. In addition, the left lateral orbital wall was dislocated to the posterior-lateral side (Figure 2A). In a brain CT scan, she had signs of left subarachnoid hemorrhage that explained the cause of consciousness disturbance (Figure 2B).

**Figure 3:**
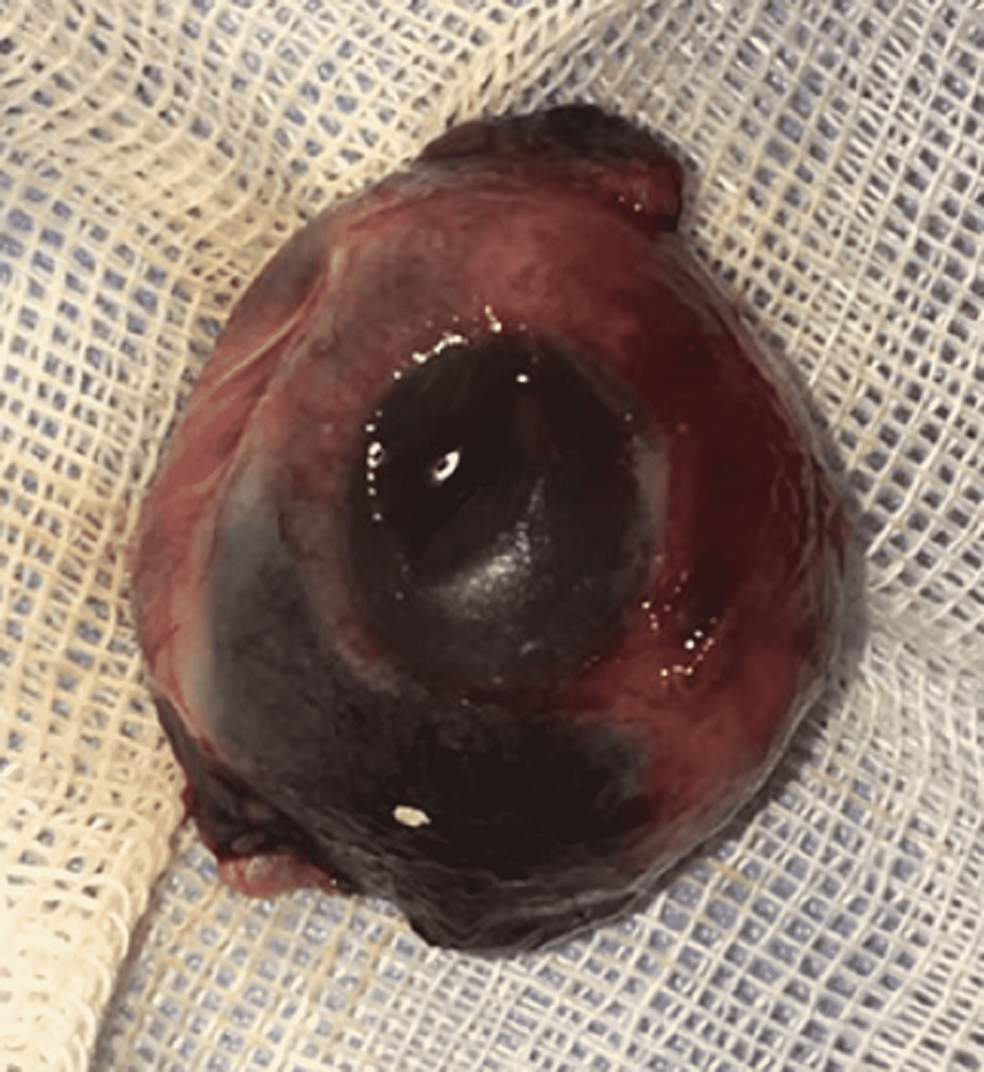
The removed necrotic and auto-enucleated globe

Due to the life-threatening condition with decreased arousal level, she was admitted to the ICU for further interventions. As the conservative and temporary ophthalmic intervention until stabilizing her general condition, the avulsed globe was reduced into the orbital cavity, and tarsorrhaphy was performed to avoid re-displacement of the avulsed globe. After 24 h, the auto-enucleated globe became necrotic due to the discontinuation of the globe's vascular supplies during the trauma. The patient underwent close follow-up visits to detect any signs of sympathetic ophthalmia in the fellow eye, but fortunately, these signs were not present in that eye. Finally, after stabilizing her general condition, the globe, the remnant of necrotic extraocular muscles, and a segment of the avulsed optic nerve were removed in the operation theater (Figure 3).

Other traumatized and necrotic tissues of the orbital cavity were debrided, too. Finally, the remnant of tenon’s capsule and conjunctiva were closed. Then, she was treated with intravenous antibiotics (including Vancomycin 500 mg three times a day and Ceftazidime 1 g twice a day) for three days and discharged from the ophthalmology service with oral ciprofloxacin and prednisolone 50 mg/day tapered gradually and advised for the future reconstructive orbital surgeries including implantation of ocular prosthesis. Despite our suggestion, she did not come back for these interventions.

## Discussion

Here, we reported an unusual case of traumatic enucleation following head trauma after falling with no significant orbital wall disassembly following blunt head injury, contrary to the most cases of traumatic enucleation that were associated with significant and multiple orbital wall fractures following direct and high-energy traumas [5,8-14]. In this case, we hypothesized that the patient was probably predisposed to the globe luxation including having shallow orbit and/or lax extraocular muscles. She did not have any significant past ocular or systemic history. During the blunt trauma to the temporal side of her head, the orbital cavity volume was reduced suddenly with an unexpected increase in the orbital pressure. Subsequently, the nasal sidewall plays the role of a fulcrum (as described for one of the mechanisms of globe luxation by Morris et al. [10]) and finally, the globe is displaced outwardly with the dehiscence of lax structures including the optic nerve and extraocular muscles.

Avulsion of the globe can be classified as incomplete when just the optic nerve is avulsed and complete when all extraocular muscles and optic nerve are disrupted (considered traumatic enucleation) [11]. For incomplete cases of globe avulsion, the preference is the maintenance of the globe, unless the globe's vascular supplies are disrupted [15]. But, for complete globe avulsion (traumatic enucleation), most surgeons suggest the removal of the globe and adjacent components because the functional and anatomical rehabilitation of the globe is impossible, but it also increases the risk of sympathetic ophthalmia (a serious vision-threatening complication that is caused by a T-cell-mediated autoimmune reaction to exposed ocular antigens of the injured eye) progression to the fellow eye [11,12,16]. Other indications for globe removal in these cases with an intact globe are no probability of globe salvage according to the ophthalmologist's judgment and progression of any signs of globe necrosis [8,13,14]. But, some investigators suggest postponing the globe removal after the psychological rehabilitation of the patients. In addition, the scleral tissue of the retained globe could also be used to implant ocular prosthesis in future reconstructive orbital surgeries [14]. In this case, the patient had complete globe avulsion with early signs of globe necrosis progression, but her general condition did not allow us to remove the avulsed globe immediately. Therefore, we examined her closely for any signs of sympathetic ophthalmia progression to the uninvolved eye, and after stabilizing her general condition, due to a non-salvageable eye, she underwent the globe, remnant of necrotic extraocular muscles, and part of the optic nerve removal.

In patients with traumatic enucleation, the reconstruction of the anophthalmic socket for further interventions including implantation of an ocular prosthesis is completely difficult and challengeable, especially when the conjunctival and the orbital tissues are missing [14].

## Conclusions

Traumatic enucleation is a rare condition without known treatments. It can be caused not only by direct and high-energy traumas, but also by the indirect mode of trauma with no significant orbital wall disassembly. In these patients, predisposition to globe luxation must be considered and patients should be advised to protect the fellow eye from any direct or indirect head and ocular trauma.
